# Determination of Cholesterol Content in Butter by HPLC: Up-to-Date Optimization, and In-House Validation Using Reference Materials

**DOI:** 10.3390/foods9101378

**Published:** 2020-09-29

**Authors:** Lukáš Kolarič, Peter Šimko

**Affiliations:** Faculty of Chemical and Food Technology, Institute of Food Science and Nutrition, Slovak University of Technology in Bratislava, Radlinského 9, 812 37 Bratislava, Slovakia; qsimko@stuba.sk

**Keywords:** cholesterol, butter, saponification, extraction, HPLC, optimization, validation

## Abstract

This work deals with up-to-date optimization of cholesterol content determination when saponification and extraction procedures as well as HPLC conditions were studied. As found, optimal conditions for saponification process were identified by 15 min heating in the presence of 0.015 L of methanolic KOH solution with a concentration 1 mol/L with subsequent 0.015 L n-hexane–chloroform binary mixture (1:1, *v*/*v*) double extraction. HPLC separation consisted of isocratic elution with flow rate of 0.5 mL/min mobile phase composed of acetonitrile/methanol 60:40 (*v*/*v*) and stationary phase Zorbax Eclipse Plus C18 column 2.1 × 100 mm, 3.5 μm particle size diameters with detector wavelength 205 nm. The method passed through in-house validation criteria and its suitability was verified by analysis of butter reference materials. In final, the average content of cholesterol content in butter was determined at 2271.0 mg/kg. Thus, the method is suitable for the determination of cholesterol content in butter and probably also in other dairy products.

## 1. Introduction

Dairy products are complex foods containing many essential components necessary for human health and full-body vitality. However, elevated milk and dairy products consumption can result in heart diseases due to saturated fatty acids content, especially through the mechanism of increased blood lipids and total cholesterol and/or low-density lipoproteins content [1]. Cholesterol is the most important animal sterol to be found in foods of animal origin such as milk, eggs, meat, fish, and their products [2]. Cholesterol accounts for 0.25–0.40% of the total lipids contained in raw milk and it is present in fat globule membranes, fat core itself, as well as complexed with milk proteins particularly in skimmed milk [3]. In addition to its essential roles in human health, elevated human plasma cholesterol content may increase the risk of cardiovascular diseases and atherosclerosis; therefore, a maximum intake of cholesterol of 300 mg per day for adults has been recommended by professional associations [4]. However, according to the dietary guidelines advisory committee report [5], available evidence shows no appreciable relationship between the consumption of dietary cholesterol and blood serum cholesterol [5]. According to the recent European Society of Cardiology (ESC) and European Atherosclerosis Society (EAS) guidelines for the management of dyslipidaemias, published in 2019, the key initiating event in the atherogenesis is the retention of low-density lipoprotein cholesterol (LDL-C) and other cholesterol-rich apolipoprotein containing lipoproteins within the arterial wall. Therefore, it is recommended that very high-risk patients should achieve an LDL-C level of <55 mg/dL (<1.4 mmol/L) and at least a 50% reduction from baseline LDL-C levels [6]. As around 20–25% of cholesterol in our body comes from food, so it is important to know its content in our dietary intake [7].

Butter is a water-in-oil emulsion, generally containing a minimum of 80 g milk fat/100 g and a maximum of 16 g water/100 g [8]. Nowadays, besides traditional butter, various butter products such as reduced-fat and low-fat butter (spreads) have been developed and commercialized to meet the public health concerns improve butter qualities [8]. According to the report of Food and Agriculture Organization of the United Nations (FAO) about the overview of global dairy market developments in 2018, global butter exports expanded by 7.5% [9], and according to OECD-FAO Agricultural Outlook (2019), the average consumption of butter in Europe between the years 2016–2018 achieved 3.5 kg/capita [10], which is one of the highest value worldwide. Since butter is rich in milk fat, which increases total blood and LDL-cholesterol levels, the butter consumption is often associated with an increased risk of cardiovascular diseases [11]. As butter is one of the most consumed milk products worldwide, there is an obvious requirement for the monitoring of cholesterol content by appropriate analytical methods. 

Currently, the official methods for the determination of cholesterol content in milk containing emulsified foods are the AOAC (Association of Official Analytical Chemists) and IDF (International Dairy Federation). The IDF method is primarily used for dairy products, such as raw milk, infant formula, cream, and cheese, and it is an accurate, precise, and stable method [12]. In general, the methods for cholesterol content determination in food can be divided into three major categories: classical chemical methods based on the Abell–Kendall protocol, fluorometric and colorimetric enzymatic assays, and analytical instrumental approaches such as gas and liquid chromatography [7]. The choice of a suitable method depends mainly on the food matrices that are going to be analyzed. For example, in processed foods containing primarily lipids of vegetable origin, the enzymatic method lacks specificity because other sterols with a 3β-OH group including phytosterols can also be oxidized and form similar pigments [13]. The most appropriate and frequently used steps for the sample preparation before liquid chromatography include the direct saponification followed by the extraction of the unsaponifiable residue into the nonpolar solvent [14]. Direct saponification has been preferred due to possibility to convert nonpolar fatty acid esters to polar products with their following effective removal by multiple extraction with n-hexane [14,15,16,17]. The other options can be single extraction by toluene [13], or three-stage extraction with diethyl ether [18]. Besides that, a mixture of polar and nonpolar solvents has been proposed to obtain better cholesterol extraction from various food matrix where cholesterol is usually bound by many other biological compounds such as lipoproteins, proteins, and phospholipids [13].

So, it is clear that saponification and the extraction process are crucial in cholesterol content determination by HPLC. Therefore, the aim of work was up-to-date optimization of saponification and extraction procedures from the point of duration time and solvent choice, or consumption, respectively. Additionally, HPLC conditions and in-house validation of the developed method using reference materials were included to obtain, in final, up-to-date method applicable for determination of the cholesterol content in butter.

## 2. Materials and Methods 

### 2.1. Standards and Reagents

All reagents and standards were of analytical grade. Cholesterol standard was purchased from Sigma-Aldrich with a purity ≥99%, potassium hydroxide from Mikrochem (Pezinok, Slovakia), chloroform, n-hexane, toluene, ethanol, and sodium sulfate anhydrous from Centralchem s.r.o. (Bratislava, Slovakia), methanol and acetonitrile (HPLC grade) from Fisher Chemical (Loughborough, UK). Reference materials, butter mild soured (muva-BU-1311), sweet cream butter (muva-BU-1312), and sweet cream butter salted (muva-BU-1314) were obtained from Muva Kempten GmbH (Kempten, Germany). Eleven commercially available butter samples were bought in the local markets. Butter samples consisted of 9 samples with the declared fat content 82%, one sample with 82.5%, and one with 84% declared fat content.

### 2.2. Sample Preparation

The saponification process was performed according to our previous study with slight modifications [17]. During the optimization of the saponification process, 0.5 g of samples were refluxed with various volumes of methanolic solution of 1 mol/L KOH (0.005, 0.007, 0.010, 0.012, and 0.015 L) during different saponification times (15, 30, 45, and 60 min). The extraction process was performed with the various extraction solvents (n-hexane, chloroform, toluene, and the mixture of n-hexane and chloroform (1:1, *v*/*v*)) in a one, two, and three numbers of extraction. Total volume of solvent used for single extraction was 0.015 L. For increasing the polarity of saponifiable residue, 10 mL of deionized water was added. To avoid the formation of emulsion during the extraction, 1 mL of 96% (*v*/*v*) ethanol was added to the saponified matter. Then, the combined extracts were filtrated through anhydrous Na_2_SO_4_, and evaporated using a rotary vacuum evaporator (Heidolph, Germany) until dry; the residue was dissolved in 5 mL of methanol, solution-filtered using syringe PTFE filter with 0.2 μm membrane (Agilent Technologies, Santa Clara, CA, USA), and analyzed by HPLC. 

### 2.3. Instrument and Chromatographic Conditions

HPLC analysis was performed using an Agilent Technologies 1260 infinity system (USA) equipped with a vacuum degasser, a quarterly pump, an autosampler, and the UV-DAD detector was set at 205 nm. Isocratic elution was performed at a flow rate of 0.5 mL/min using the mobile phase consisted of acetonitrile/methanol 60:40 (*v*/*v*). The injection volume was 10 µL and the temperature was set at 30 °C. As a stationary phase, Zorbax Eclipse Plus C_18_ column (2.1 × 100 mm, 3.5 μm particle size, Agilent, Santa Clara, CA, USA) was used with the guard column Zorbax SB-C_18_ (4.6 × 12.5 mm, 5 μm particle size, Agilent, Santa Clara, CA, USA). Total run time of analysis was 7 min with retention time of cholesterol in 5.6 min. The results were recorded using the OpenLab CDS software, ChemStation Edition for LC, and LC/MS systems (product version A.01.08.108).

### 2.4. Calibration Curve

A stock solution of cholesterol (1000 mg/L) was prepared by dissolving 25 mg of cholesterol in 25 mL of methanol. Then, 10 working standard solutions were prepared from the stock solution to obtain the concentrations of 2, 6, 10, 25, 40, 50, 75, 100, 300, and 350 mg/L. Each solution was analyzed in quadruple and average peak areas were calculated.

### 2.5. Method Validation

The developed method was validated according to IUPAC technical report [19] and Eurachem guide for the fitness for purpose of analytical methods [20]. The following parameters were determined: selectivity, limit of detection (LOD), limit of quantification (LOQ), linearity, precision, trueness, accuracy, and ruggedness. 

The selectivity is the ability to discriminate between the analyte or species to be determined and other materials in test sample [19]; it was tested for eventual co-eluting impurities and spectral interferences during HPLC analysis by comparison of the scanned UV spectrum of the cholesterol standard and cholesterol in samples, identified by external standard addition procedure.

The LOD and LOQ were calculated as 3 or 10 times the standard deviation of ten blanks divided by slope of the calibration curve [4]. 

For the determination of linearity and working range of the method, the calibration curve was recorded by plotting of the measuring signal on the y-axis against the known quantity of the cholesterol standard on the x-axis [19]. The linearity was described by the correlation coefficient for linear regression (r). The value of r close to 1.0 indicates smaller dispersion of the experimental points and greater reliability of the estimated regression coefficients [15].

According to [20], the accuracy of the method is studied as trueness and precision. Trueness was evaluated as the closeness of agreement between a test result and the reference value in terms of bias. The bias was recorded in two approaches: 1. analysis of reference materials, and 2. recovery experiment using spiked samples with cholesterol standard in three different concentrations (500, 1000, and 1500 mg/L). The bias was expressed as a relative recovery (R%) by the ratio of the mean value of the test sample (*n* = 12) and the reference value. The precision was determined as the closeness of agreement between independent test results obtained under stipulated conditions. The precision of the method was characterized by the repeatability and intermediate precision obtained over a longer period within the same laboratory. The repeatability was investigated by injecting four replicates of samples in quadruple on the same day. The intermediate precision was evaluated on three different days by preparing four replicates from the same sample on each day. The precision was then evaluated from the standard deviation (SD) and relative standard deviation (RSD). For indicating the acceptability of the method with respect to reproducibility, the Horwitz ratio (HorRat) was also calculated as the ratio of the observed RSD to the corresponding predicted relative standard deviation calculated from the Horwitz equation, PRSD_R_ (%) = 2C^−0.15^, where C is the concentration expressed as a mass fraction [21]. 

The ruggedness of the method has been characterized as the resistance to results’ changes of an analytical method when any deviation was made in experimental conditions [19]. While ruggedness can be considerably affected by the quality of stationary phase, changes in retention time, LOD, LOQ, and the content of cholesterol in the sample was studied using analytical columns with different parameters as follows: 1. 2.1 × 50 mm, 3.5 μm particle size, 2. 4.6 × 50 mm, 3.5 μm particle size, and 3. 2.1 × 50 mm, 5 μm particle size. 

### 2.6. Statistical Analysis

Results are expressed as mean ±standard deviation or as a percentage. Statistical analysis was performed using Microsoft Excel version 365. The obtained data were subjected to one-way analysis of variance (ANOVA) and to the Student´s test, and the values were considered significantly different when *p* < 0.05.

## 3. Results and Discussion

### 3.1. Optimization of the Sample Preparation

The sample preparation for the cholesterol determination in dairy matrices consists of two crucial steps: the saponification and the extraction. The earlier studies had also included lipid extraction from the sample before saponification, but as described by Dinh et al. [22], this step was later eliminated to reduce the analysis time and solvent use. The extraction of cholesterol from the saponified sample seems to be the critical step as various studies used various solvents and numbers of extraction with, of course, different results. It is clear that the selection of extraction solvent should be based on its immiscibility with the aqueous phase and without any effects on chromatographic elution of cholesterol [23]. Thus, the first goal was to identify the most suitable solvent and the number of extractions. For this purpose, the butter sample was used. Three various solvents with different polarities were tested: n-hexane, toluene, and chloroform due to the fact that the n-hexane was the choice in most previous studies because of its polarity, which is suitable for extracting the cholesterol from the aqueous environment. 

**Figure 1:**
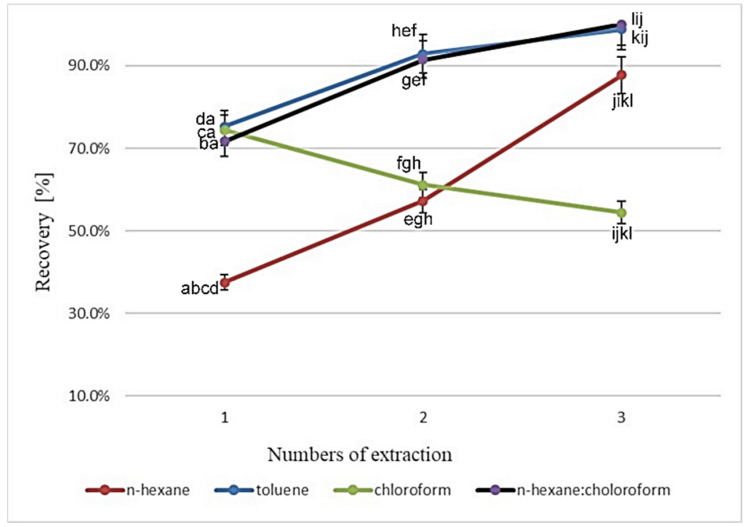
The effect of extraction numbers on the cholesterol recovery from butter sample applying various solvents: n-hexane, toluene, chloroform and the binary mixture of n-hexane and chloroform Different letters indicate significant difference at *p* < 0.05.

However, there are different numbers of extractions to be applied for cholesterol extraction in literature. For example, Albuquerque et al. [16] extracted cholesterol from various foods two times. On the contrary, Borkovcová et al. [14] or Bauer et al. [15] used just single extraction while Dias et al. [24] or Ramalho et al. [25] applied triple extraction. Indeed, single extraction is not really appropriate enough from the point of recovery. As can be seen in Figure 1, the efficiency of single n-hexane extraction reached only 37.5%. This finding is in close accordance with studies that suggested triple n-hexane extraction, but the recovery of cholesterol according to our measurement was not as high as by using toluene. The results obtained by n-hexane extraction differ significantly at *p* < 0.05 in comparison to other extraction solvents. The advantages of n-hexane over toluene are less toxicity and better vacuum evaporating, but the strength of cholesterol extraction was not satisfying very much. As follows from Figure 1, better results were found using the toluene as an extraction solvent. A single extraction by toluene was also used by Dinh et al. [13] with high recovery ranging from 94% to 102%. Almost the same recoveries (99–104%) were reported by Lin et al. [26] during the extraction of cholesterol from cow milk. Probably due to its higher polarity compared to n-hexane, the toluene was able to extract more cholesterol from the saponified sample. The difference between the results obtained in double and triple extraction was not significantly different at *p* < 0.05 while the difference between results obtained in single and double extraction was significantly different thus it can be suggested at least double extraction with toluene for appropriate results. The use of toluene has, however, some disadvantages: it is a highly flammable and toxic solvent with a higher boiling point than n-hexane, which extends the time of evaporation. Besides that, the use of toluene is prone to the formation of emulsions, which could lead to either decreased recovery or overestimated cholesterol concentration because the losses of toluene and cholesterol into the emulsions might not be proportional [13]. Chloroform has the highest polarity in comparison to n-hexane and toluene and is less flammable and toxic than toluene. However, chloroform as a single solvent is not suitable for cholesterol extraction from a saponified matrix, because it brings about the extraction of many other polar molecules, thus the selectivity of this extraction is not enough. Daneshfar et al. [23] concluded that the extraction of cholesterol with chloroform does not provide a separable liquid/liquid system. However, it is notable (Figure 1) that the recovery of cholesterol extracted in a single step by chloroform was still higher than in single extraction with n-hexane. Due to the information mentioned above, the fourth extraction procedure was performed with n-hexane/chloroform mixture (1:1, *v*/*v*). As follows from Figure 1, the application of this mixture exhibited practically almost the same recovery in comparison with toluene. The recoveries obtained by these two extractions were not significantly different at *p* < 0.05. Besides that, the results showed better reproducibility, i.e., lower values of relative standard deviations and the difference between the results obtained in double and triple extractions were not significantly different at *p* < 0.05. So, it could be thus concluded that double extraction with the chloroform and n-hexane mixture is more effective than with n-hexane, and more convenient than with toluene due to better evaporating properties and less toxicity. The repeatability of the method was also better and more precise in comparison with single n-hexane or toluene, respectively. Application of other binary solvent mixtures is mentioned in the literature, e.g., Adu et al. [27] extracted cholesterol from samples in triple extraction using a mixture of diethyl ether and water (5:2), Bertolín et al. [4] applied a mixture of n-hexane:ethyl acetate 9:1, and Chen et al. [28] realized three times extraction with a n-hexane:petroleum ether mixture (50:50, *v*/*v*). 

**Figure 2:**
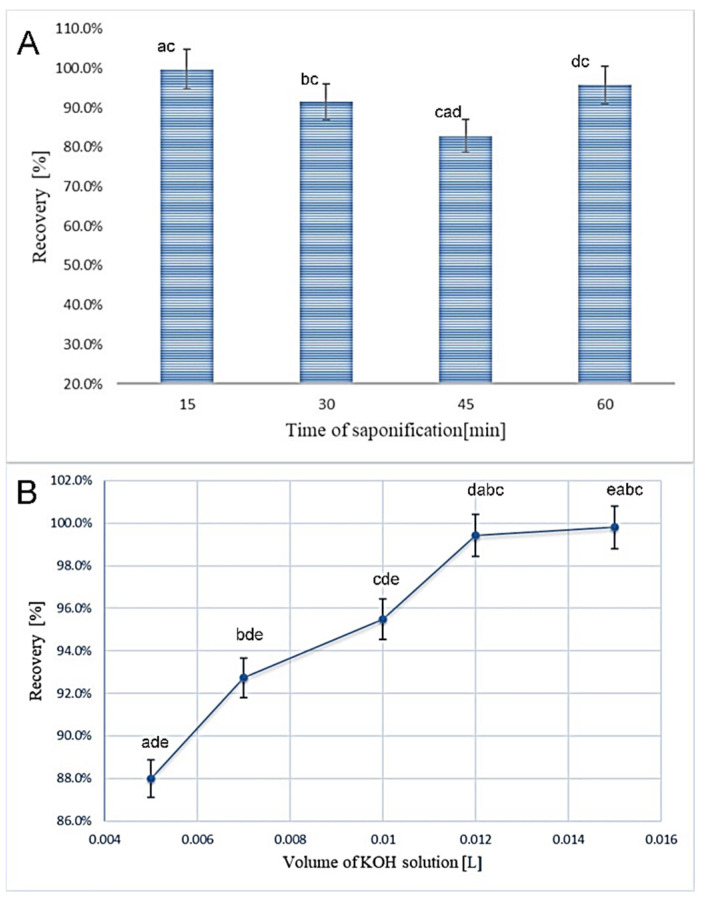
The effect of time of saponification (**A**) and the volume of KOH solution to cholesterol recovery (**B**) from butter sample. Different letters on top indicate significant difference at *p* < 0.05.

The saponification process is essential for the separation of cholesterol from other unsaponifiable components. There are two types of saponification, the indirect (requires a previous Folch extraction) and the direct one [16]. Direct saponification is chosen to simplify sample preparation procedure and most studies showed that direct saponification has superior recovery and accuracy compared with conventional lipid extraction and saponification [29]. Therefore, direct saponification was also chosen in these experiments when the optimization process was directed to the duration of saponification and the volume of methanolic KOH solution (1 mol/L) needed for saponification at mild boiling of the reaction mixture (Figure 2). Although the difference between cholesterol recovery in the samples saponified 15, 30, and 60 min was not significant at *p* < 0.05, saponification lasting 15 min resulted in the highest cholesterol recovery, thus this time was adopted as sufficient, i.e., optimal (Figure 2A). On the contrary, the volume of a methanolic KOH solution influenced considerably cholesterol recovery, as follows from Figure 2B. The highest recoveries were obtained in the presence of 12 and 15 mmol of KOH, besides that, the results were not significantly different at *p* < 0.05. It means that 12 mL of the solution was necessary for complete cholesterol release from esterified complexes. According to Dinh et al. [13], the most suitable conditions are ethanolic KOH solution with a concentration of 0.33–0.5 mol/L with saponification temperatures ranging from 55 to 75 °C lasting 60 min. Oh et al. [18] or Tahir et al. [30] used for saponification of milk 10% ethanolic KOH, and the test tube heated at 70 °C for 30 min. Ramalho et al. [25] applied the same time and temperature conditions but different KOH concentration (50%). Lin et al. [26] achieved the best results with the same concentration of KOH as applied in this study but with a longer time of saponification (30 min). Different results were also described by Albuquerque et al. [16] when 5 mL of 0.4 mol/L ethanolic KOH were applied during 30 min. Saponification time 15 min was used by López-Cervantes et al. [31]. Bauer et al. [15] described cold saponification of milk samples with 8 mL of aqueous KOH solution (50%) and 12 mL of ethyl alcohol addition. The disadvantage of the proposed method was, however, a long time of saponification lasting up to 22 h. The saponification conditions are thus very different in various studies. It clearly demonstrates how saponification, together with extraction of unsaponifiable residue, is a critical step in the analysis of cholesterol content in dairy products. Moreover, conditions of saponification and extraction are also influenced by the food matrices. In general, cholesterol in food is present in two forms, free and esterified. If the food matrices contain more cholesterol in esterified forms, such as eggs, the saponification will require a longer time and higher concentration of KOH solution [32]. 

### 3.2. Method Validation

The in-house validation process was made on the three kinds of butter reference materials at the same conditions, i.e., saponification time 15 min, volume of methanolic KOH solution (1 mol/L) 15 mL, and the extraction solvent mixture of n-hexane/chloroform (1:1, *v*/*v*). The results of this section are summarized in the Table 1.

The selectivity of the method was confirmed by scanning of UV spectra of cholesterol eluted during HPLC. As follows from Figure S3, both spectra (cholesterol standard and cholesterol in real sample analysis) are practically identical, with the match almost to 1 (0.99) of purity peak parameter ratio what confirms sufficiently absence of any interfering substance in eluted cholesterol peak. 

The linearity of the method was approved by the calibration curve, which was linear over the range of 2–380 mg/kg with the correlation coefficient *r* = 0.999. LOD and LOQ values were 3.6 and 11.8 mg/kg, which are similar to Albuquerque et al. [16] who reached values 3 and 11 mg/kg, respectively. In the other study, higher values of LOD (11.10 mg/kg) and LOQ (33.65 mg/kg) was observed by Bauer et al. [15]. According to them, the differences are coming from the different methods employed for the sample preparation. As stated by Osman and Chin [33], HPLC provides lower LOD and LOQ in comparison to the Lieberman–Bouchard spectrophotometric method and gas chromatography, which agrees also to our previous study [17].

The accuracy of the method was studied as trueness and precision. Measurement of trueness is an expression of how close the mean of an infinite number of results is to a reference value [20]. In the first approach, the trueness of our method was established by the comparison of the results with the reference values of three kinds of sweet cream butter. The certified values of cholesterol content for sweet cream butter salted (muva-BU-1314), butter mild soured (muva-BU-1311), and sweet cream butter (muva-BU-1312) were 2276 ± 135, 2334 ± 211, and 2337 ± 196 mg/kg, respectively. The obtained relative recoveries varied from 101.4 to 102.5%. These data confirm that the method was enabled to reach equal recovery. In the second approach, the recovery was studied by spiking with cholesterol at different concentration levels 500, 1000, and 1500 mg/L, respectively. In addition, this approach resulted in high recoveries ranging from 103.9 to 106.3%. According to Ahn et al. [12], the values in the spiking test ranged from 98.1 to 102.3%. The slightly lower recoveries were obtained by Bertolín et al. [4], ranging from 94.7 to 97.7%. The other parameter, which is necessary for the determination of method accuracy is the precision. The precision was determined according to IUPAC technical report [19] as the closeness of agreement between independent test results obtained under stipulated conditions. According to Bauer et al. [15], RSD values of up to 15% are acceptable, although a maximum variation of 5% for micro constituents is recommended. The RSD values of repeatability and intermediate precision of our method ranged from 0.6 to 3.9% and 0.8 to 1.8%, respectively. The reproducibility of the method could be also evaluated by the Horwitz equation. The reproducibility is approved when the Horrat parameter is less or equal 2, at maximum [17,34]; the Horrat parameter is also an essential part of validation procedures to be used for the adoption of official analytical methods for food quality applied by the European Commission [35]. In all three reference materials, the Horrat value was 0.01, which indicates excellent method precision. 

The last important validation parameter is the ruggedness of the method. The ruggedness or robustness of an analytical procedure is a measure of its capacity to remain unaffected by small, but deliberate variations in method parameters [20]. The robustness of the proposed method was studied using different parameters of the HPLC column. The results are summarized in Table 2. The change in the length of the column (from 100 mm to 50 mm) caused a decrease in retention time, and LOD and LOQ values (3.0 mg/kg and 10.0 mg/kg, respectively). The LOD and LOQ achieved the higher values in a column with 5 µm particle size, and with higher thickness of column (4.6 mm). Thus, the columns with 3.5 µm particle size had better sensitivity. In summary, it was not noticed any significant changes in cholesterol content on analyzed samples, and differences among the results were not significantly different at *p* < 0.05. The RSD was only 1.9%, which indicates that the proposed method is not influenced by parameters of analytical columns. 

### 3.3. Analysis of the Butter 

The validated method was applied for the determination of cholesterol content in 11 kinds of butter and results are summarized in Table 3. The cholesterol content in butter samples varied from 1983.7 to 2582.8 mg/kg with a mean value of 2271.0 mg/kg and a median of 2247.5 mg/kg. According to Derewiaka et al. [36], the cholesterol content in butter is between 2043 and 3824 mg/kg. The cholesterol content in various samples of butter purchased in Polish supermarkets ranging from 1768 to 2648 mg/kg with the mean value of 2407 mg/kg, which is slightly higher than in this work. Gonçalves and Baggion [37] determined the cholesterol content in butter from 1928 to 2263 mg/kg, while Seçkin et al. [38] between 2512.7 and 3690.4 mg/kg. 

## 4. Conclusions

Due to the headway in the area of food analysis, the HPLC method for cholesterol content determination in butter has been updated and individual steps optimized. As found, optimal conditions for the saponification process comprise 15 min heating of 0.5 g butter in the presence of 0.015 L of methanolic KOH solution with concentration 1 mol/L. Additionally, the extraction procedure of cholesterol from saponified matter was optimized when 0.015 L of n-hexane-chloroform binary mixture (*v*/*v*) exhibited high extraction effectivity during double extraction. HPLC separation consisted of isocratic elution with a flow rate of 0.5 mL/min mobile phase composed of acetonitrile/methanol 60:40 (1:1, *v*/*v*) and stationary phase Zorbax Eclipse Plus C_18_ column 2.1 × 100 mm, 3.5 μm particle size diameters with set detector wavelength 205 nm. At given conditions, chromatographic separation of cholesterol was not affected by any coeluting impurity. The method passed through in house validation criteria and its suitability was verified by analysis of three kinds of butter reference materials. As found, experimentally obtained data were in close accordance with the data declared by reference materials provider. In conclusion, average content in the samples of butter purchased in Slovakian markets was determined at 2271.0 mg/kg. Thus, the method is suitable as an operative up-to-date method for the determination of cholesterol content in butter and probably also in other dairy products.

## Figures and Tables

**Table 1:** Method validation (linearity, recovery, and precision of method).

**Method linearity**				
Slope ^a^	11.348 ± 0.026			
Intercept ^a^	13.514 ± 3.908			
Correlation coefficient (r)	0.999 ± 0.000			
LOD [mg/kg]	3.6			
LOQ [mg/kg]	11.8			
**Method recovery** ^b^				
Reference material	Reference value [mg/kg]	Determined value [mg/kg]	Recovery [%]	RSD [%]
muva-BU-1312	2337 ± 196	2395.6 ± 72.6	102.5 ± 0.0	3.0
muva-BU-1311	2334 ± 211	2381.6 ± 66.6	102.0 ± 0.0	2.8
muva-BU-1314	2276 ± 135	2307.7 ± 58.9	101.4 ± 0.0	2.6
The amount of cholesterol standard spiked to the butter sample [mg/L]	Determined value [mg/kg]	Recovery [%]	RSD [%]	
0	2367.0 ± 29.9	-	-	
500	2460.6 ± 39.0	103.9 ± 0.0	1.6	
1000	2475.4 ± 31.5	104.6 ± 0.0	1.3	
1500	2515.9 ± 52.7	106.3 ± 0.0	2.1	
**Method precision** ^c^				
Reference material	Repeatability	RSD [%]	Intermediate precision [RSD%]	Horrat
muva-BU-1312	Day 1 Day 2 Day 3	2.5 2.6 2.2	1.8	0.01
muva-BU-1311	Day 1 Day 2 Day 3	3.9 2.3 0.6	0.9	0.01
muva-BU-1314	Day 1 Day 2 Day 3	1.4 3.5 1.9	0.8	0.01

The values are expressed as mean ± standard deviation, ^a^ *n* = 4, ^b^ *n* = 12, ^c^ *n* = 4; muva-BU-1312, sweet cream butter; muva-BU-1311, butter mild soured; muva-BU-1314, sweet cream butter salted; LOD, limit of detection; LOQ, limit of quantification; RSD, relative standard deviation.

**Table 2:** The ruggedness of proposed method.

Dimension of Column(Zorbax Eclipse Plus C_18_)	Retention Time [min]	Slope	Intercept	LOD [mg/kg]	LOQ [mg/kg]	Cholesterol Content [mg/kg] ^a^
2.1 × 100 mm, 3.5 µm particle size	5.6	11.348	13.514	3.6	11.8	2367.0 ± 29.9
2.1 × 50 mm, 3.5 µm particle size	2.2	11.349	17.320	3.0	10.0	2360.4 ± 27.9
4.6 × 50 mm, 3.5 µm particle size	4.4	5.713	30.071	5.5	18.4	2289.3 ± 53.8
2.1 × 50 mm, 5.0 µm particle size	2.2	11.480	69.452	6.8	22.5	2417.5 ± 92.6

^a^ The values are expressed as mean ± standard deviation; LOD, limit of detection; LOQ, limit of quantification. Muva-BU-1311, butter mild soured was used for evaluation the method ruggedness.

**Table 3:** Cholesterol content of butter samples.

Sample	Cholesterol Content [mg/kg]	RSD [%]
1	2323.1 ± 29.4	1.3
2	2241.1 ± 97.9	4.4
3	2582.8 ± 36.5	1.4
4	2201.6 ± 52.3	2.4
5	1983.7 ± 52.5	2.7
6	2252.3 ± 117.5	5.2
7	2272.3 ± 87.0	3.8
8	2215.9 ± 4.4	0.2
9	2460.0 ± 30.3	1.2
10	2247.5 ± 119.1	5.3
11	2200.8 ± 24.7	1.1

The values are expressed as mean ± standard deviation. The fat content of samples is as follow: sample No. 1–9 = 82% declared fat content, sample No.10 = 84% declared fat content and sample No.11 = 82.5% declared fat content.

## Supplementary Materials

The following are available online at https://www.mdpi.com/2304-8158/9/10/1378/s1, Figure S1: 3D plot of cholesterol standard record obtained by UV-DAD detector; Figure S2: 3D plot of cholesterol in butter sample obtained by UV-DAD detector; Figure S3: Spectra of cholesterol scanned during HPLC analysis—red curve is the spectrum of cholesterol standard while blue curve is the spectrum of cholesterol scanned during butter analyses.

## References

[1] Pereira P.C. (2014). Milk nutritional composition and its role in human health. Nutrition.

[2] Gorassini A., Verardo G., Fregolent S.-C., Bortolomeazzi R. (2017). Rapid determination of cholesterol oxidation products in milk powder based products by reversed phase SPE and HPLC-APCI-MS/MS. Food Chem..

[3] Shingla K.M., Mehta B.M. (2018). Cholesterol and its oxidation products: Occurrence and analysis in milk and milk products. Int. J. Health Anim. Sci. Food Saf..

[4] Bertolín J.R., Joy M., Rufino-Moya P.J., Lobón S., Blanco M. (2018). Simultaneous determination of carotenoids, tocopherols, retinol and cholesterol in ovine lyophilised samples of milk, meat, and liver and in unprocessed/raw samples of fat. Food Chem..

[5] Dietary Guidelines Advisory Committee (2015). Scientific Report of the 2015 Dietary Guidelines Advisory Committee: Advisory Report to the Secretary of Health and Human Services and the Secretary of Agriculture.

[6] ESC/EAS Guidelines (2020). 2019 ESC/EAS Guidelines for the management of dyslipidaemias: Lipid modification to reduce cardiovascular risk. Eur. Heart J..

[7] Li L.-H., Dutkiewicz E.P., Huang Y.-C., Zhou H.-B., Hsu C.-C. (2019). Analytical methods for cholesterol quantification. J. Food Drug Anal..

[8] Lee C.-L., Liao H.-L., Lee W.-C., Hsu C.-K., Hsueh F.-C., Pan J.-Q., Chu C.-H., Wei C.-T., Chen M.-J. (2018). Standards and labelling of milk fat and spread products in different countries. J. Food Drug Anal..

[9] Food and Agriculture Organization of the United Nations Overview of global dairy market developments in 2018. http://www.fao.org/publications/card/en/c/CA3879EN/.

[10] (2019). OECD-FAO Agricultural Outlook 2019–2028.

[11] Lovegrove J.A., Givens D.I. (2016). Dairy food products: Good or bad for cardiometabolic disease?. Nutr. Res. Rev..

[12] Ahn J.H., Jeong I.-S., Kwak B.M., Leem D., Yoon T., Yoon C., Jeong J., Park J.M., Kim J.M. (2012). Rapid determination of cholesterol in milk containing emulsified foods. Food Chem..

[13] Dinh T.T.N., Thompson L.D., Galyean M.L., Brooks J.C., Patterson K.Y., Boylan L.M. (2011). Cholesterol Content and Methods for Cholesterol Determination in Meat and Poultry. Compr. Rev. Food Sci. Food Saf..

[14] Borkovcová I., Janoušková E., Dračková M., Janštová B., Vorlová L. (2009). Determination of Sterols in Dairy Products and Vegetable Fats by HPLC and GC Methods. Czech. J. Food Sci..

[15] Bauer L.C., de Santana D.A., dos Macedo M.S., Torres A.G., de Souza N.E., Simionato J.I. (2014). Method Validation for Simultaneous Determination of Cholesterol and Cholesterol Oxides in Milk by RP-HPLC-DAD. J. Braz. Chem. Soc..

[16] Albuquerque T.G., Oliveira M.B.P.P., Sanches-Silva A., Costa H.S. (2016). Cholesterol determination in foods: Comparison between high performance and ultra-high performance liquid chromatography. Food Chem..

[17] Kolarič L., Šimko P. (2020). The comparison of HPLC and spectrophotometric method for cholesterol determination. Potravinárstvo Slovak J. Food Sci..

[18] Oh H.I., Shin T.S., Chang E.J. (2001). Determination of Cholesterol in Milk and Dairy Products by High-Performance Liquid Chromatography. Asian Australas J. Anim. Sci..

[19] IUPAC Technical Report (2002). Harmonised guidelines for the in-house validation of methods of analysis. IUPAC/In-House/Budapest.

[20] Eurachem (2014). The Fitness for Purpose of Analytical Methods—A Laboratory Guide to Method Validation and Related Topics.

[21] Horwitz W., Albert R. (2006). The Horwitz Ratio (HorRat): A Useful Index of Method Performance with Respect to Precision. J. AOAC Int..

[22] Dinh T.T.N., Blanton J.R., Brooks J.C., Miller M.F., Thompson L.D. (2008). A simplified method for cholesterol determination in meat and meat products. J. Food Compos. Anal..

[23] Daneshfar A., Khezeli T., Lofti H.J. (2009). Determination of cholesterol in food samples using dispersive liquid-liquid microextraction followed by HPLC-UV. J. Chromatogr. B.

[24] Dias H.M.A.M., Berbicz F., Pedrochi F., Baesso M.L., Matioli G. (2010). Butter cholesterol removal using different complexation methods with beta-cyclodextrin, and the contribution of photoacoustic spectroscopy to the evaluation of the complex. Food Res. Int..

[25] Ramalho H.M.M., Casal S., Oliveira M.B.P.P. (2011). Total Cholesterol and Desmosterol Contents in Raw, UHT, Infant Formula Powder and Human Milks Determined by a New Fast Micro-HPLC Method. Food Anal. Methods.

[26] Lin Y.-T., Wu S.-S., Wu H.-L. (2007). Highly sensitive analysis of cholesterol and sitosterol in foods and human biosamples by liquid chromatography with fluorescence detection. J. Chromatogr. A.

[27] Adu J.K., Amengor D.K., Kabiri N., Orman E., Patamia S.A.G., Okrah B.K. (2019). Validation of a Simple and Robust Liebermann-Burchard Colorimetric Method for the Assay of Cholesterol in Selected Milk Products in Ghana. Int. J. Food Sci..

[28] Chen Y.Z., Kao S.Y., Jian H.C., Yu Y.M., Li J.Y., Wang W.H., Tsai C.W. (2015). Determination of cholesterol and four phytosterols in foods without derivatization by gas chromatography-tandem mass spectrometry. J. Food Drug Anal..

[29] Shazamawati Z.H., Alina A.R., Mashitoh A.S., Juhana M.J.T. (2013). Cholesterol Oxidation Products Analysis in Meat and Poultry. Middle-East. J. Sci. Res..

[30] Tahir M.N., Kwon C., Jeong D., Cho E., Paik S.R., Jung S. (2013). Cholesterol reduction from milk using β-cyclodextrin immobilized on glass. J. Dairy Sci..

[31] López-Cervantes J., Sánchez-Machado D.I., Ríos-Vázquez N.J. (2006). High-performance liquid chromatography method for the simultaneous quantification of retinol, α-tocopherol, and cholesterol in shrimp waste hydrolysate. J. Chromatogr. A.

[32] Hansen H., Wang T. (2015). Does the Saponification-GC Method Underestimate Total Cholesterol Content in Samples Having Considerable Cholesterol Esters?. J. Am. Oil Chem. Soc..

[33] Osman H., Chin Y.K. (2006). Comparative sensitivities of cholesterol analysis using GC, HPLC and spectrophotometric methods. Malays. J. Anal. Sci..

[34] Ribeiro T.M., Brandäo P.R.G. (2017). Development and Validation of Graphitic Carbon Analysis of Graphite Ore Samples. Tecnol. Metal. Mater. Min..

[35] (2011). Commission regulation (EU) No 836/2011 of 19 August 2011 amending Regulation (EC) No 333/2007 laying down the methods of sampling and analysis for the official control of the levels of lead, cadmium, mercury, inorganic tin, 3-MCPD and benzo(a)pyrene in foodstuffs. Off. J. Eur. Union.

[36] Derewiaka D., Sosińska E., Obiedziński M., Krogulec A., Czaplicki S. (2011). Determination of the adulteration of butter. Eur. J. Lipid Sci. Technol..

[37] Gonçalves M.F.D., Baggio S.R. (2012). Evaluation of quality of butter from different provenance. Food Sci. Technol..

[38] Seçkin A.K., Gursoy O., Kinik O., Akbulut N. (2005). Conjugated linoleic acid (CLA) concentration, fatty acid composition and cholesterol of some Turkish dairy products. LWT Food Sci. Technol..

