# Supplementary figures and images for: Determination of Cholesterol Content in Butter by HPLC: Up-to-Date Optimization, and In-House Validation Using Reference Materials
Source: Foods. 2020 Sep 29;9(10):1378. doi: 10.3390/foods9101378 (PMC7650802; doi:10.3390/foods9101378)

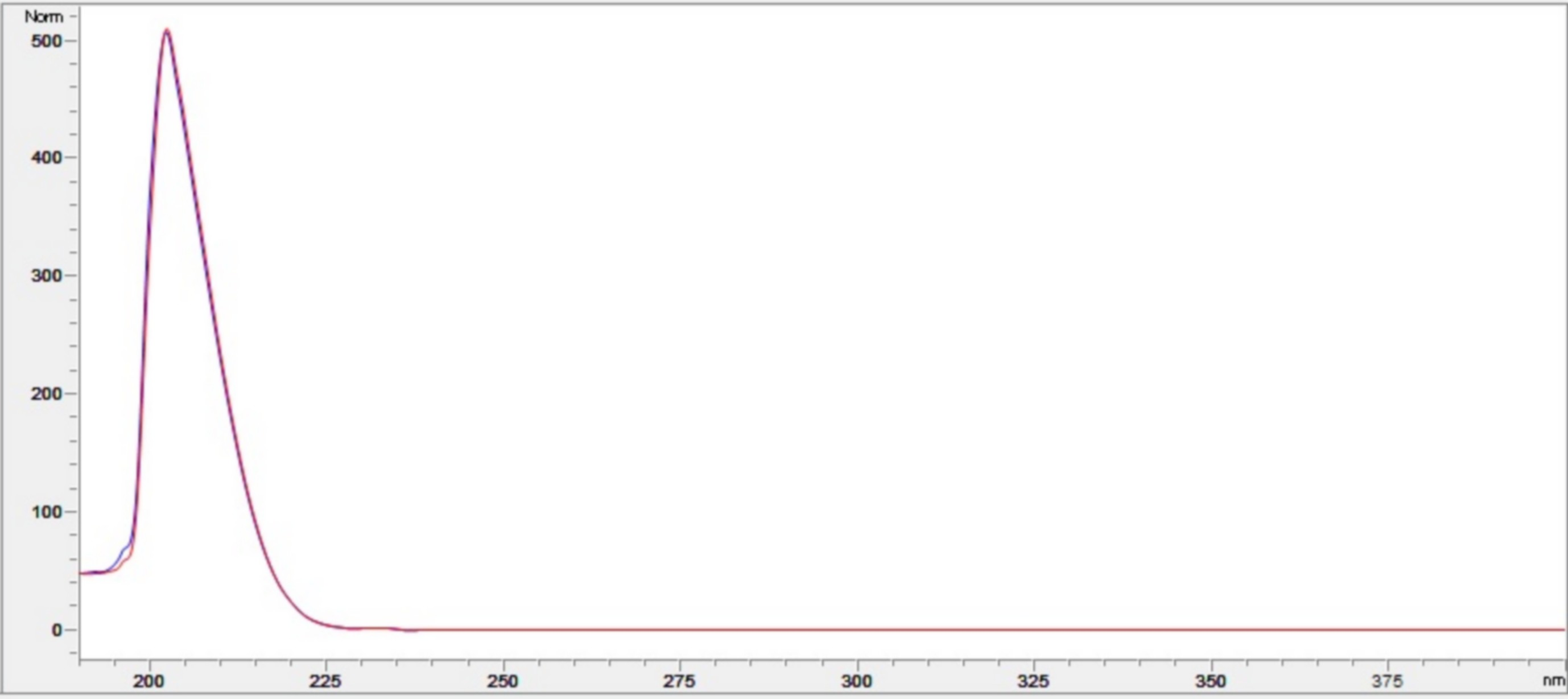

Supplement: Supplementary file 1 [file foods-09-01378-s001.zip › S3 Spectra of cholesterol scanned during HPLC analysis.png]

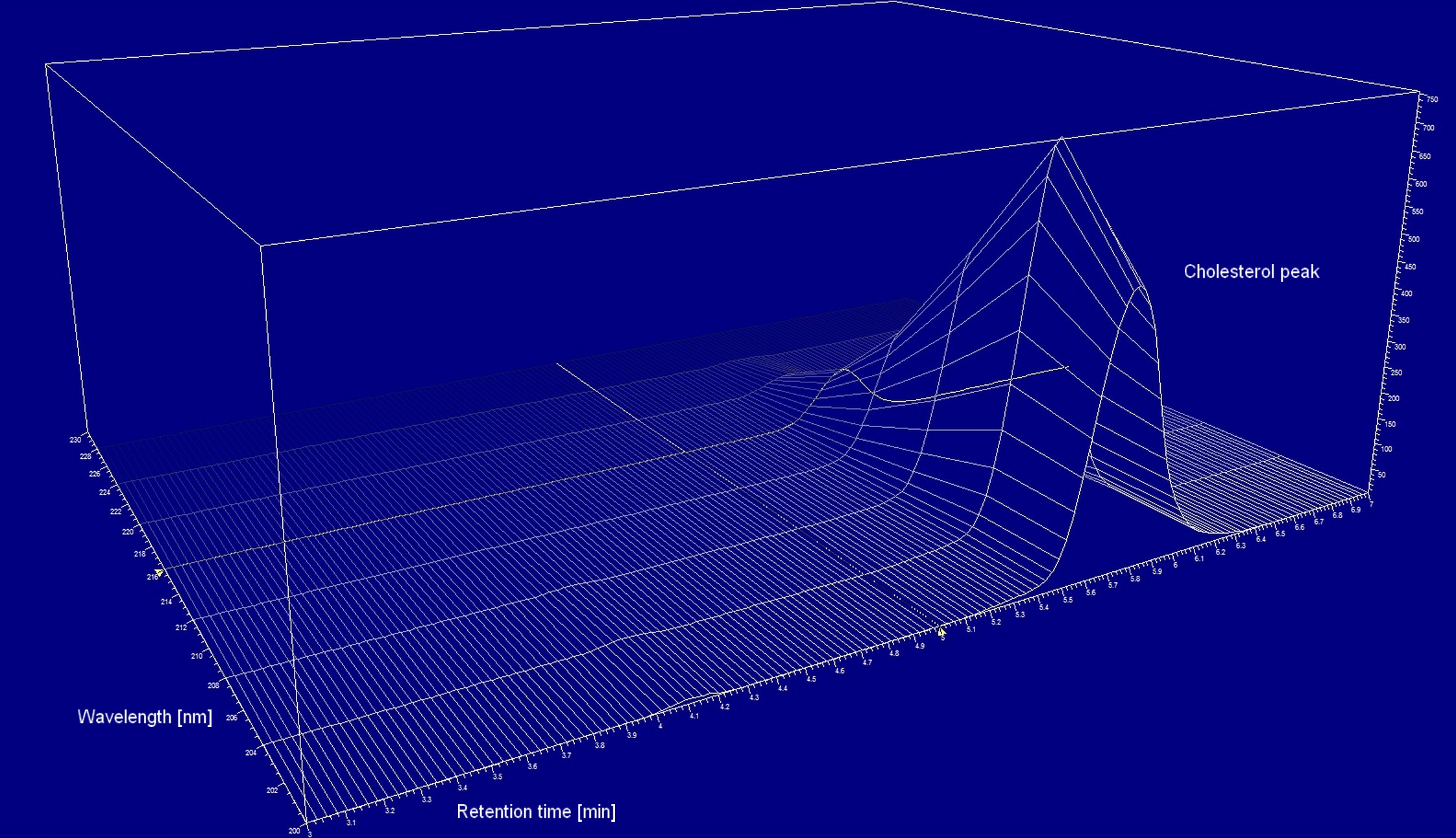

Supplement: Supplementary file 1 [file foods-09-01378-s001.zip › S1 3d plot cholesterol standard.jpg]

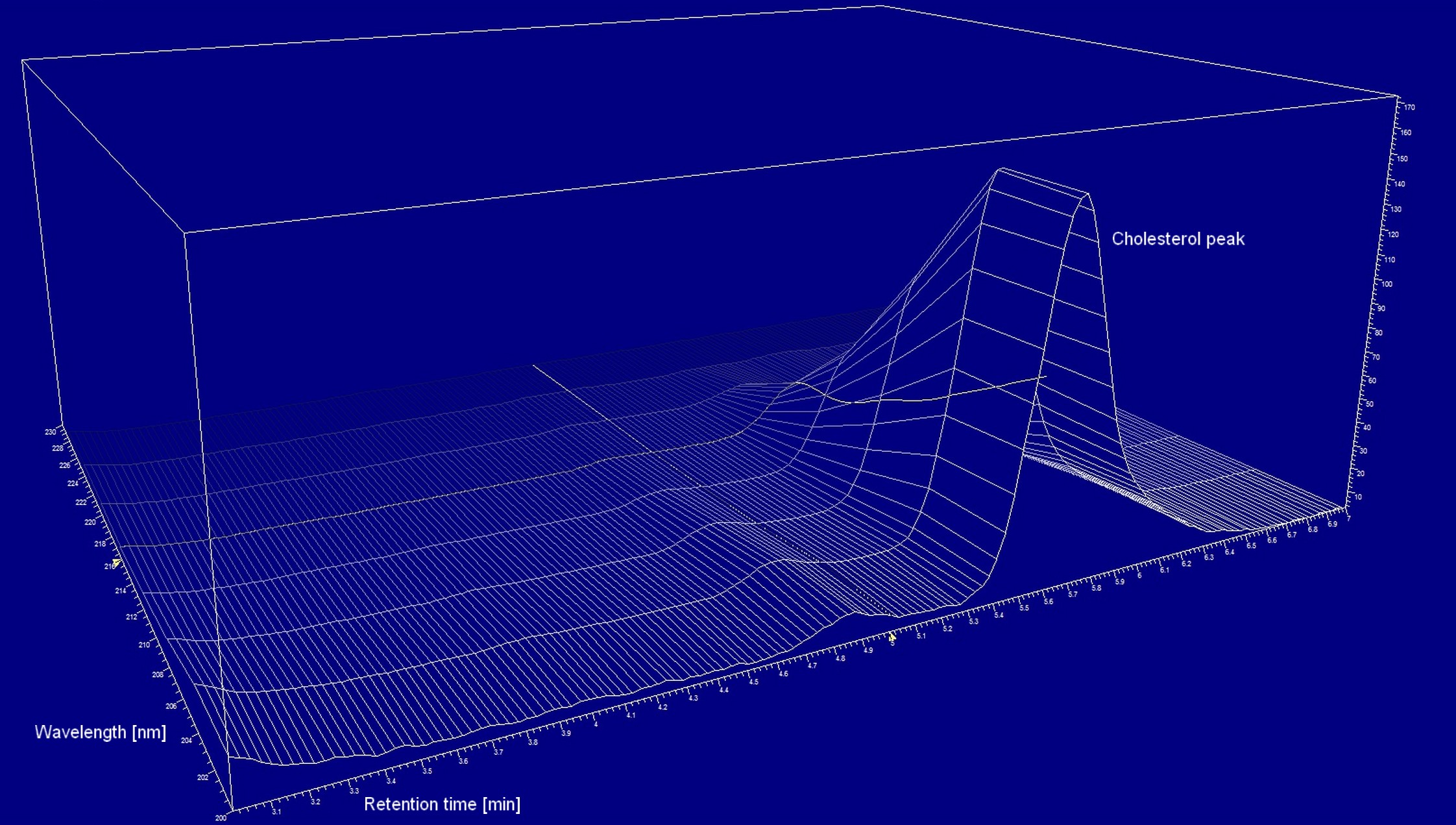

Supplement: Supplementary file 1 [file foods-09-01378-s001.zip › S2 3D plot cholesterol butter.jpg]
